# Association between baseline serum uric acid and development of LDL-C level in patients with first acute myocardial infarction

**DOI:** 10.1186/s12872-021-02383-x

**Published:** 2021-11-30

**Authors:** Yang Chen, Congcong Ding, Longlong Hu, Yuehua Ruan, Kai Zou, Cong Dai, Yanhui Liao, Hanhui Liao, Yi Xia, Yuanbin Zhao, Renqiang Yang

**Affiliations:** 1 Department of Cardiovascular Medicine, The Second Affiliated Hospital of Nanchang University, No. 1 Minde Road, Nanchang of Jiangxi, 330006 China; 2 Department of Cardiovascular Medicine, The First Affiliated Hospital of Nanchang University, Nanchang of Jiangxi, China

**Keywords:** Uric acid, Low-density lipoprotein cholesterol, Acute myocardial infarction

## Abstract

**Background:**

Data on the relationship of baseline serum uric acid (SUA) with development of low-density lipoprotein cholesterol (LDL-C) level in patients with first acute myocardial infarction (AMI) are limited. The present study is to evaluate whether elevated SUA predicts the development of LDL-C in the first AMI.

**Methods:**

This is a retrospective 6-month cohort study of 475 hospitalized Chinese patients who underwent first AMI between January 2015 and December 2019 and were reevaluated half a year later at the Department of Cardiology, the Second Affiliated Hospital of Nanchang University, Jiangxi Province, China. The associations of baseline SUA with the percentage decrease of LDL-C (%) and LDL-C control were analyzed by using logistic regression analyses, multivariate linear regression analyses and the restricted cubic spline.

**Results:**

Over the 6-month follow-up, baseline SUA was independently and positively associated with the percentage decrease of LDL-C (%) and LDL-C control in a dose response fashion. After multivariable adjustment, per SD increment of baseline SUA (120.58 μmol/L) was associated with 3.96% higher percentage decrease of LDL-C(%). The adjusted OR (95% CI) for LDL-C control was 5.62 (2.05, 15.36) when comparing the highest tertile (SUA ≥ 437.0 μmol/L) to the lowest tertile (< 341.7 μmol/L) of baseline SUA.

**Conclusions:**

Among Chinese patients with first AMI, higher baseline SUA was associated with higher LDL-C deduction percentage (%), and higher rate of LDL-C control in the short-term follow-up, respectively. SUA acquired when AMI occurred was prone to be profitable in predicting the risk stratification of uncontrolled LDL-C and dyslipidemia management.

**Supplementary Information:**

The online version contains supplementary material available at 10.1186/s12872-021-02383-x.

## Background

High low-density lipoprotein cholesterol (LDL-C) is a strong risk factor for cardiovascular diseases[1]. Reducing LDL-C levels is advocated to prevent and control atherosclerotic cardiovascular disease (ASCVD) hazards, especially in high risk ASCVD patients, such as patients with myocardial infarction. It is necessary to adjust the lipid-lowering therapy according to the curative effect of the patient in clinical practice. Therefore, to identify the risk factors for predicting the rate of LDL-C reduction or the probability of LDL-C control is clinically relevant. Growing evidence validated the relationship of serum uric acid (SUA) with metabolic syndrome[2], cardiometabolic diseases[3], cardiovascular prognosis[4], diabetes and prediabetes[5]. Notably, an increasing number of studies have indicated the association between SUA and dyslipidemia. Some cross-sectional studies have reported that hyperuricemia is primarily associated with hypertriglyceridemia, low high-density lipoprotein cholesterol (HDL-C) and non-HDL-C[6–10]. Further, a retrospective cohort study found that elevated SUA increases the risk for developing high LDL-C, as well as hypertriglyceridemia[11]. However, no studies investigated whether SUA predicts the change of LDL-C in patients with first acute myocardial infarction (AMI). The presented study was designed to explore the relation between SUA and changes of LDL-C level in patients with first AMI.

## Methods

### Study design and population

This was a single-center, retrospective, longitudinal study that was carried in 'real-world' conditions via an observational design. We collected all patients diagnosed as myocardial infarction between January 2015 and December 2019 in the Department of Cardiology, the Second Affiliated Hospital of Nanchang University, Jiangxi Province, China. Then, by analyzing their medical records, we screened out the standard–compliant patients. Inclusion criteria were as follows: patients were diagnosed as AMI firstly and reevaluated half a year later in the Second Affiliated Hospital of Nanchang University. The exclusion criteria were included: a history of coronary heart disease, cerebral infarction, hyperlipidemia and other diseases that require lipid-lowering drugs, malignancy, thyroid function disease, liver failure, kidney failure or any other systemic disease known to be associated with secondary dyslipidemias, pregnancy or lactation; treatment with corticosteroids therapy within the 6-month period before enrollment.

### Data collection and follow up

Previous medical history, age, sex, body mass index (BMI), blood pressure (BP) condition in hospital, smoke status, drink status, conditions of AMI and medication information for all subject were obtained by trained doctors or nurses. The laboratory data of the initial examination of every subject were also collected. Follow-up visits were scheduled at 6 months after the initiation of the AMI described below. Plasma samples were processed within two hours of collection and analyzed by an automatic biochemical analyzer (AU5400; Olympus) using commercially available kits.

### Diagnosis of AMI

AMI was defined as a rise in cardiac biomarkers with at least one value above the 99th percentile of the upper reference limit of the assay with evidence of myocardial ischemia including any of the following: ischemic symptoms, ischemic changes on electrocardiography, evidence of loss of viable myocardium on imaging, or angiographic evidence of coronary atherothrombosis. Information on AMI type (ST-segment elevation myocardial infarction (STEMI), non-ST-segment elevation myocardial infarction (NSTEMI)) was documented in the patients’ medical records.

### Definitions

Patients’ hospital medical records were used as baseline indicators and blood samples were collected within 24 h. Percentage decrease in LDL-C (%) was defined as the LDL-C at baseline minus the LDL-C obtained at the 6-month follow-up and then divided by the LDL-C at baseline. LDL-C control was defined when patients’ LDL-C of < 1.4 mmol/L and LDL-C reduction of ≥ 50% from baseline according to 2019 ESC/EAS Guidelines for the management of dyslipidaemias.

### Statistical analysis

Baseline characteristics are listed as mean ± Standard Deviation (SD) or the median (25th percentile-75th percentile) for continuous variables and proportions (%) for categorical variables by SUA tertiles. Differences in baseline characteristics were compared using Student’s t-test, Mann–Whitney test or chi-square tests, accordingly. Further, the Holm-Bonferroni stepdown procedure for multiple comparisons was used to control the type I error rate [12]. Linear regression was performed to examine the relationship of baseline SUA and percentage decrease in LDL-C (%) with adjustments for the above baseline covariates. Logistic regression models were performed to determine the relationship of SUA tertiles (< 341.7, 341.7–437.0, and ≥ 437.0 μmol/L) with LDL-C control after adjustment for pertinent variables. Possible effect modifications of the association between SUA and LDL-C control were investigated by stratified analyses. Interactions were examined by including interaction terms in the regression models. In addition, we explored the relation of baseline SUA with the percentage decrease in LDL-C (%) and LDL-C control using restricted cubic spline (smooth fitting curve).

All the data analyses were performed using R version 3.4.3 (www.R-project.org) and EmpowerStates (www.empowerstats.com). A 2-sided *P* < 0.05 was considered statistically significant.

### Ethical considerations

This study was approved by the Regional Ethics Committee of the Second Affiliated hospital of Nanchang University, and the approval number was No. [2014] 059. The patients signed a consent for the processing of personal data. The authors fully took the safety and fairness principle into account. The data are anonymous, and we didn’t harm the participants and protected the privacy right of the participants.The preceding procedures were conducted in accordance with the principles of the Declaration of Helsinki.

## Results

### Study participants and baseline characteristics

**Fig. 1:**
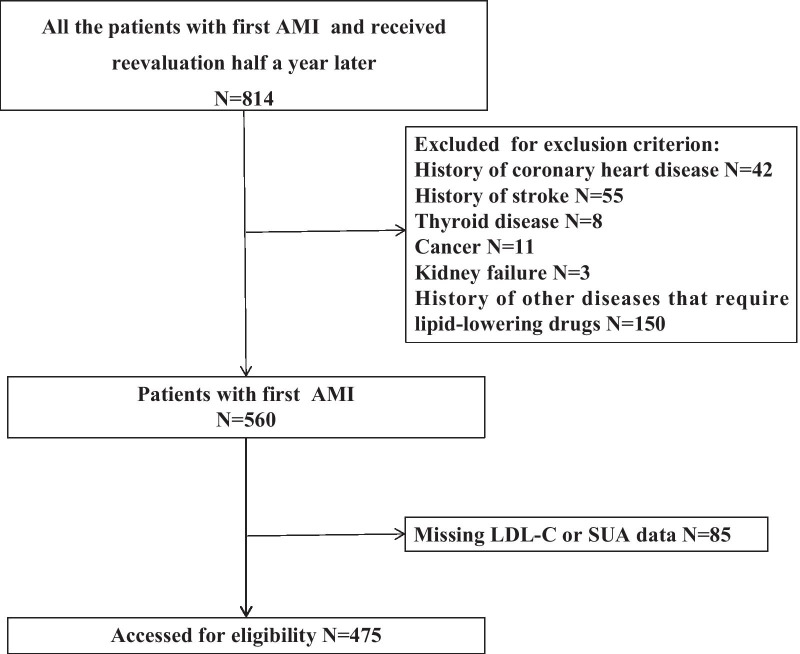
Flow chart of participants

There were 475 patients with first AMI who were hospitalized in the Department of Cardiology of the Second Affiliated Hospital of Nanchang University between January 2015 and December 2019 and underwent medical examination at the center half a year later. We excluded 42 subjects with history of coronary, 55 subjects with stroke, 8 subjects with hyperthyroidism or hypothyroidism, 11 subjects with cancer, 3 subjects with kidney failure and 150 subjects had other diseases that already receive lipid-lowering therapy. There were 85 patients among the standard–compliant patients who were missing important data at the baseline(Fig. 1). Participant characteristics by baseline SUA are presented in Table 1. The mean baseline serum uric acid level was 401.87 ± 119.42umol/L. The prevalence of hyperuricemia (> 420 μmol/L) was 38.95%. Patients with high level of SUA tend to have a higher rate of males, smoke, use of diuretics, higher blood urea nitrogen (BUN), creatinine (CRE), estimated glomerular filtration rate (eGFR) during the admission and lower apolipoprotein A (ApoA), lower HDL-C, higher rate of LDL-C control after 6 months (all *P* < 0.05 after Holm-Bonferroni correction). Table 2 presented the conditions of AMI in the population by SUA tertiles.

**Table 1:** Characteristics of the study participants by SUA tertiles

Variables	Total	SUA, μmol/L			*P*	*P*^a^
		Tertile 1 (< 341.7)	Tertile 2 (≥ 341.7, < 437.0)	Tertile 3 (≥ 437.0)		
N	475	158	158	159		
Age, years	63.00 (54.00–72.00)	65.00 (56.00–73.00)	62.00 (54.00–70.75)	63.00 (53.00–72.00)	0.556	NS
Male, n (%)	371 (78.11%)	102 (64.56%)	131 (82.91%)	138 (86.79%)	< 0.001	< 0.001
BMI, kg/m^2^	24.09 (22.37–26.56)	24.43 (22.73–26.77)	24.03 (22.43–26.39)	23.72 (22.19–26.08)	0.191	NS
SBP, mm Hg	124.04 ± 21.42	124.15 ± 19.77	124.02 ± 22.70	123.96 ± 21.82	0.997	NS
DBP, mm Hg	75.45 ± 14.09	74.94 ± 12.37	76.40 ± 14.63	75.00 ± 15.16	0.583	NS
Smoke, n (%)	226 (47.58%)	61 (38.61%)	79 (50.00%)	86 (54.09%)	0.017	0.047
Drink, n (%)	145 (30.53%)	38 (24.05%)	56 (35.44%)	51 (32.08%)	0.078	NS
Diabetes, n (%)	93 (19.58%)	37 (23.42%)	24 (15.19%)	32 (20.13%)	0.179	NS
Hypertension, n (%)	250 (52.63%)	90 (56.96%)	77 (48.73%)	83 (52.20%)	0.339	NS
Gout, n (%)	11 (2.32%)	2 (1.27%)	6 (3.80%)	3 (1.89%)	0.296	NS
*Laboratory results*						
TBIL, μmol/L	14.12(10.99–19.54)	13.39 (10.87–18.07)	14.65 (11.11–20.12)	14.31 (11.00–19.73)	0.532	NS
DBIL, μmol/L	3.22 (2.26–4.62)	3.45 (2.25–4.94)	3.29 (2.38–4.62)	3.00 (2.15–4.28)	0.091	NS
IBIL, μmol/L	10.87 (8.06–15.23)	10.31 (7.33–14.01)	11.50 (8.75–15.43)	11.03 (8.17–15.43)	0.588	NS
ALT, U/L	31.72 (19.73–51.16)	28.52 (20.68–43.70)	30.79 (18.68–49.22)	35.16 (20.05–56.09)	0.314	NS
AST, U/L	40.70 (25.00–86.34)	44.50 (25.40–91.82)	35.97 (23.61–82.30)	43.82 (27.45–76.03)	0.815	NS
BUN, mmol/L	6.14 (5.00–7.92)	5.69 (4.91–6.73)	6.27 (4.80–7.85)	6.97 (5.36–9.28)	< 0.001	< 0.001
CRE, μmol/L	82.95 (69.05–100.41)	73.19 (62.70–83.78)	83.43 (69.70–97.10)	94.19 (79.96–118.69)	< 0.001	< 0.001
eGFR, mL · min^−1^ · 1.73 m^−2^	82.54 (65.42–100.38)	90.30 (74.83–109.60)	83.88 (70.01–101.75)	69.80 (50.84–86.82)	< 0.001	< 0.001
TC, mmol/L	4.44 (3.85–5.38)	4.31 (3.81–5.07)	4.55 (3.92–5.38)	4.59 (3.90–5.47)	0.997	NS
TG, mmol/L	1.43 (1.04–1.97)	1.29 (0.97–1.74)	1.48 (1.08–1.96)	1.54 (1.10–2.12)	0.638	NS
LDL-C, mmol/L	2.80 (2.19–3.42)	2.59 (2.13–3.14)	2.89 (2.17–3.59)	2.90 (2.31–3.42)	0.333	NS
HDL-C, mmol/L	1.00 (0.85–1.17)	1.03 (0.86–1.21)	0.99 (0.85–1.14)	0.96 (0.84–1.13)	0.244	NS
ApoA, g/L	0.96 (0.82–1.10)	0.98 (0.82–1.12)	0.96 (0.85–1.14)	0.94 (0.81–1.08)	0.370	NS
ApoB, g/L	0.86 (0.71–1.03)	0.81 (0.69–0.98)	0.87 (0.70–1.05)	0.88 (0.72–1.05)	0.395	NS
*Medication use, n (%)*						
Statin use					0.356	
ATF10mg/ROS5mg	3 (0.63%)	0 (0.00%)	1 (0.63%)	2 (1.26%)		NS
ATF20mg/ROS10mg	283 (59.58%)	97 (61.39%)	87 (55.06%)	99 (62.26%)		NS
ATF40mg/ROS20mg	185 (38.95%)	59 (37.34%)	70 (44.30%)	56 (35.22%)		NS
β receptor blocker	421 (88.63%)	146 (92.41%)	136 (86.08%)	139 (87.42%)	0.175	NS
Diuretics	185 (38.95%)	45 (28.48%)	69 (43.67%)	71 (44.65%)	0.004	0.025
Uric-acid-lowering drugs	8 (1.68%)	0 (0.00%)	4 (2.53%)	4 (2.52%)	0.132	NS
*Reevaluation of cholesterol*						
TC (mmol/L)	3.47 (2.98–4.06)	3.57 (3.07–4.13)	3.54 (2.98–4.17)	3.26 (2.88–3.93)	0.039	NS
TG(mmol/L)	1.26 (0.94–1.75)	1.23 (0.92–1.69)	1.25 (0.94–1.79)	1.30 (0.96–1.77)	0.954	NS
ApoA(g/L)	1.01 (0.87–1.17)	1.09 (0.92–1.23)	1.01 (0.88–1.14)	0.97 (0.81–1.12)	0.012	0.030
ApoB(g/L)	0.61 (0.51–0.78)	0.64 (0.55–0.79)	0.62 (0.52–0.79)	0.57 (0.48–0.71)	0.162	NS
HDL-C(mmol/L)	1.03 (0.88–1.22)	1.06 (0.93–1.32)	1.04 (0.88–1.18)	0.98 (0.83–1.16)	0.004	0.014
LDL-C(mmol/L)	1.82 (1.46–2.29)	1.88 (1.59–2.24)	1.81 (1.47–2.36)	1.71 (1.34–2.29)	0.050	NS
LDL-C control, n (%)	49 (10.32%)	8 (5.06%)	13 (8.23%)	28 (17.61%)	< 0.001	0.006

Data are expressed as mean ± standard deviation or median (interquartile range) and numbers (percentage) as appropriate

SUA, serum uric acid; BMI, body mass index; SBP, systolic blood pressure; DBP, diastolic blood pressure; STEMI, ST elevation myocardial infarction; TBil, total bilirubin; DBIL, direct Bilirubin; IBIL, Indirect Bilirubin; ALT, alanine aminotransferase; AST, aspartate aminotransferase; BUN, blood urea nitrogen; CRE, creatinine; eGFR, estimated glomerular filtration rate; TC, total cholesterol; TG, triglyceride; HDL-C, high-density lipoprotein cholesterol; LDL-C, low-density lipoprotein cholesterol; ApoA, apolipoprotein A; ApoB, apolipoprotein B; ATF, atorvastatin; ROS, rosuvastatin; NS, not significant

^a^Holm-Bonferroni-corrected *P* value

**Table 2:** Conditions of AMI in the population by SUA tertiles

Variables	Total	SUA, μmol/L			*P*	*P*^a^
		Tertile 1 (< 341.7)	Tertile 2 (≥ 341.7, < 437.0)	Tertile 3 (≥ 437.0)		
N	475	158	158	159		
Time first symptom/admission, hours	15.00 (5.00–72.00)	21.00 (5.00–48.00)	12.00 (4.25–72.00)	12.00 (5.00–72.00)	0.397	NS
Time first symptom/SUA, hours	15.17 (5.29–72.12)	22.12 (6.02–55.91)	12.76 (5.18–72.31)	14.17 (5.27–72.29)	0.418	NS
Time first symptom/LDL-C, hours	30.08 (15.96–80.31)	34.00 (16.62–66.64)	28.48 (15.04–81.61)	29.12 (17.40–82.99)	0.385	NS
MI type and location, n (%)					0.857	
NSTEMI	133 (28.00%)	48 (30.38%)	42 (26.58%)	43 (27.04%)		NS
Anterior STEMI	144 (30.32%)	46 (29.11%)	46 (29.11%)	52 (32.70%)		NS
Non anterior STEMI	198 (41.68%)	64 (40.51%)	70 (44.30%)	64 (40.25%)		NS
Number of diseased vessels, n (%)					0.524	
0	1 (0.22%)	1 (0.65%)	0 (0.00%)	0 (0.00%)		NS
1	156 (34.44%)	56 (36.36%)	51 (34.69%)	49 (32.24%)		NS
2	141 (31.13%)	49 (31.82%)	51 (34.69%)	41 (26.97%)		NS
3	142 (31.35%)	45 (29.22%)	41 (27.89%)	56 (36.84%)		NS
4	13 (2.87%)	3 (1.95%)	4 (2.72%)	6 (3.95%)		NS
Culprit vessel, n (%)					0.561	
LAD	211 (46.68%)	65 (42.48%)	70 (47.62%)	76 (50.00%)		NS
RCA	149 (32.96%)	57 (37.25%)	44 (29.93%)	48 (31.58%)		NS
LCX	89 (19.69%)	31 (20.26%)	31 (21.09%)	27 (17.76%)		NS
LM	3 (0.66%)	0 (0.00%)	2 (1.36%)	1 (0.66%)		NS
LVEF, %	56.00 (49.00–62.25)	58.00 (51.25–63.75)	56.00 (48.00–62.50)	54.00 (48.00–61.00)	0.041	NS

Data are expressed as mean ± standard deviation or median (interquartile range) and numbers (percentage) as appropriate

SUA, serum uric acid; LDL-C, low-density lipoprotein cholesterol; STEMI, ST-segment Elevation Myocardial Infarction; NSTEMI, Non-ST-segment Elevation Myocardial Infarction; LAD, left anterior descending coronary; RCA, right coronary artery; LCX, left circumflex branch; LM, left main coronary artery; LVEF, left ventricular ejection fraction;.NS, not significant

^a^ Holm-Bonferroni-corrected *P* value

**Table 3:** Prospective association between baseline SUA and percentage decrease of LDL-C (%)

SUA, μmol/L	N	Mean ± SD	Unadjusted model		Adjusted model 1		Adjusted model 2	
			β(95%CI)	*P* value	β(95%CI)	*P* value	β(95%CI)	*P* value
SUA (per SD)	475	400.96 ± 120.58	4.00 (1.52, 6.48)	0.002	4.37 (1.79, 6.95)	0.001	3.96 (1.44, 6.48)	0.002
Tertiles								
T1 (< 341.7)	158	277.18 ± 49.36	Ref	-	Ref	-	Ref	-
T2 (≥ 341.7, < 437.0)	158	390.00 ± 26.10	6.07 (0.00, 12.14)	0.051	4.51 (− 1.31, 10.33)	0.130	4.81 (− 0.80, 10.42)	0.094
T3 (≥ 437.0)	159	534.86 ± 83.86	10.10 (4.03, 16.16)	0.001	8.91 (2.74, 15.07)	0.005	8.57 (2.64, 14.49)	0.005
*P* for trend				0.001		0.005		0.005

Model 1 was adjusted for sex, BMI, age, hypertension, diabetes, smoke, drink, types of AMI, gout, TG, LDL-C, BUN, CRE, eGFR, AST, ALT, TBIL, statin use, uric acid lowering drugs, diuretics and β receptor blocker; Model 2 was adjusted for sex, BMI, age, hypertension, diabetes, smoke, drink, types of AMI, gout, TG, LDL-C, BUN, CRE, eGFR, AST, ALT, TBIL, statin use, uric acid lowering drugs, diuretics, β receptor blocker, LVEF, culprit vessel and Killip class

SUA, serum uric acid; BMI, body mass index; AMI, acute myocardial infarction; SBP, systolic blood pressure; DBP, diastolic blood pressure; TBil, total bilirubin; DBIL, direct bilirubin; IBIL, indirect bilirubin; ALT, alanine aminotransferase; AST, aspartate aminotransferase; BUN, blood urea nitrogen; CRE, creatinine; eGFR, estimated glomerular filtration rate; TC, total cholesterol; TG, triglyceride; HDL-C, high-density lipoprotein cholesterol; LDL-C, low-density lipoprotein cholesterol; LVEF, left ventricular ejection fraction

**Table 4:** Odds ratio of LDL-C control at 6 months follow-up according to continuous or tertiles of SUA

SUA, μmol/L	N	Mean ± SD	Unadjusted model		Adjusted model 1		Adjusted model 2	
			OR(95%CI)	*P* value	OR(95%CI)	*P* value	OR(95%CI)	*P* value
SUA (per SD)	475	400.96 ± 120.58	1.83 (1.38, 2.43)	< 0.001	2.14 (1.51, 3.04)	< 0.001	2.21 (1.50, 3.26)	< 0.001
Tertiles								
T1 (< 341.7)	158	277.18 ± 49.36	1	-	1	-	1	-
T2(≥ 341.7, < 437.0)	158	390.00 ± 26.10	1.68 (0.68, 4.18)	0.263	1.86 (0.70, 4.99)	0.216	1.89 (0.67, 5.31)	0.229
T3 (≥ 437.0)	159	534.86 ± 83.86	4.01 (1.77, 9.10)	< 0.001	5.76 (2.19, 15.15)	< 0.001	5.62 (2.05, 15.36)	< 0.001
*P* for trend				< 0.001		< 0.001		< 0.001

Model 1 was adjusted for sex, BMI, age, hypertension, diabetes, smoke, drink, types of AMI, gout, TG, LDL-C, BUN, CRE, eGFR, AST, ALT, TBIL, statin use, uric acid lowering drugs, diuretics and β receptor blocker; Model 2 was adjusted for sex, BMI, age, hypertension, diabetes, smoke, drink, types of AMI, gout, TG, LDL-C, BUN, CRE, eGFR, AST, ALT, TBIL, statin use, uric acid lowering drugs, diuretics, β receptor blocker, LVEF, culprit vessel and Killip class

SUA, serum uric acid; BMI, body mass index; AMI, acute myocardial infarction; SBP, systolic blood pressure; DBP, diastolic blood pressure; TBil, total bilirubin; DBIL, direct bilirubin; IBIL, indirect bilirubin; ALT, alanine aminotransferase; AST, aspartate aminotransferase; BUN, blood urea nitrogen; CRE, creatinine; eGFR, estimated glomerular filtration rate; TC, total cholesterol; TG, triglyceride; HDL-C, high-density lipoprotein cholesterol; LDL-C, low-density lipoprotein cholesterol; LVEF, left ventricular ejection fraction

### Association between baseline SUA and the percentage decrease of LDL-C (%)

**Fig. 2:**
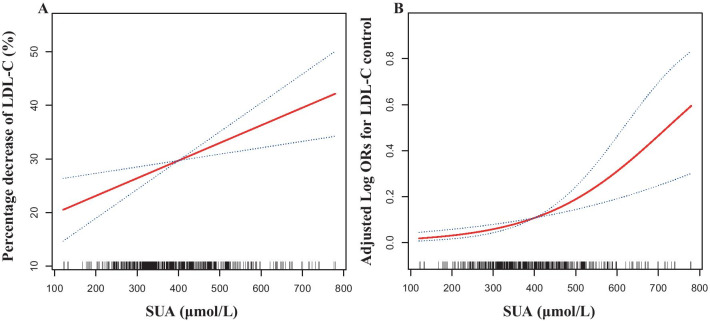
The relationship of baseline SUA with percentage decrease of LDL-C (%) (**A**), and LDL-C control at 6 months follow-up (**B**) in first AMI patients. The solid line and dashed line represent the estimated values and their corresponding 95% confidence interval. Adjustment factors included sex, BMI, age, hypertension, diabetes, smoke, drink, types of AMI, gout, TG, LDL-C, BUN, CRE, eGFR, AST, ALT, TBIL, statin use, uric acid lowering drugs, diuretics, β receptor blocker, LVEF, culprit vessel and Killip class

Table 3 shows the effects of SUA at baseline in the progression of LDL-C deduction percentage (%) in all 475 participants. Per SD increase of SUA was associated with increases of 3.96 (95% confidence interval (CI) 1.44–6.48) for the percentage decrease in LDL-C (%) after adjusting for sex, BMI, age, hypertension, diabetes, smoke, drink, types of AMI, gout, TG, LDL-C, BUN, CRE, eGFR, aspartate aminotransferase (AST), alanine aminotransferase (ALT), total bilirubin (TBIL), statin use, uric acid lowering drugs, diuretics, β receptor blocker, left ventricular ejection fraction (LVEF), culprit vessel and Killip class. Furthermore, compared with the lowest tertiles (SUA < 341.7umol/L), there was increase of 8.57 (95%CI 2.64–14.49, *P* < 0.01) for percentage decrease in LDL-C (%) in the top tertiles. Additionally, the association of baseline SUA with the percentage decrease in LDL-C (%) was likely to be linear (P for trend < 0.01). Figure 2A shows the linear relationships of baseline SUA with the percentage decrease in LDL-C (%), adjusted for sex, BMI,age, hypertension, diabetes,smoke,drink, types of AMI, gout, TG, LDL-C, BUN, CRE, eGFR, AST, ALT, TBIL, statin use, uric acid lowering drugs, diuretics, β receptor blocker, left ventricular ejection fraction (LVEF), culprit vessel and Killip class.

### SUA at baseline as a predictor of the LDL-C control at six-months follow-up

Multiple logistic regression analyses showed that the highest tertile (top tertile, ≥ 437.0) of baseline SUA had an odd ratio (OR) of 5.62 (95% CI: 2.05–15.36 *P* < 0.01) compared with the bottom tertile (first tertile, < 341.7) for LDL-C control (Table 4). Additionally, the association of baseline SUA with the LDL-C control was likely to be linear (P for trend < 0.01). Further analyses using restricted cubic spline confirmed the linearly positive association between the baseline SUA and the LDL-C control at six-months follow-up (Fig. 2B).

### Subgroup analyses

Subgroup analyses by stratification of the major covariates were performed to further confirm that the findings were reliable in the presence of potential confounders. None of the stratified variables, including sex, age, BMI, smoking, drinking, hypertension, and diabetes significantly modified the association between the baseline SUA and the condition of LDL-C control (all P for interaction > 0.05) (Additional file 1: Supplemental Figure 1).

## Discussion

In this sample of longitudinal study, baseline SUA is independently associated with the percentage decrease of LDL-C (%) and LDL-C control in first AMI patients. The restricted cubic spline indicated that the relationship of baseline SUA with the percentage decrease of LDL-C (%) and LDL-C control was linear.

To our knowledge, seldom studies have addressed the relationship between SUA and change of LDL-C level in populations with AMI underlying statin lipid-lowering therapy. Berkowitz performed a prospective study of 125 participants and found that the correlation coefficient between the triglycerides and uric acid concentrations was r = 0.64 but only r =  − 0.07 between the cholesterol and uric acid levels[8]. Another cross-sectional study of 653 patients with gout and 63 patients with asymptomatic hyperuricemia reported that HDL-C is a protective predictor of SUA levels in gout (β =  − 60.797, P = 0.013)[10]. Moreover, another population-based study of the 9580 participants undergoing routine physical examinations in urban China also observed a significant association of the serum TG with hyperuricemia both in male (AUC = 0.659, 95%CI 0.645–0.674) and in female (AUC = 0.678, 95%CI 0.665–0.690)[9]. Additionally, a post hoc data analysis[7] from NHANES III study of civilian US population reported the results concerning the association of the dyslipidemia among individuals with hyperuricemia, besides, the adjusted ORs (95% CI) were 0.29 (0.19, 0.39) mmol/L, 0.33 (0.26, 0.41) mmol/L, 0.14 (0.01, 0.27) mmol/L, − 0.08 (− 0.11, − 0.05) mmol/L, 0.09 (0.05, 0.12) g/L for the top quintiles of the TC, TG, LDL-C, HDL-C, and APOB, respectively, compared with the lowest tertiles. A retrospective 5-year cohort study of 6476 healthy Japanese adults showed that high baseline SUA was an independent risk for developing high LDL-C both in men (OR:1.159 per 1 mg/dL increase, 95%CI:1.009–1.331) and women (OR:1.215, 95%CI:1.061–1.390)[11]. Different from previous researches, our study included a retrospective longitudinal sample and further showed that baseline SUA level was independently associated with the percentage decrease in LDL-C (%) and LDL-C control in first AMI populations. Higher SUA level was significantly associated with higher LDL-C deduction percentage (%), and higher rate of LDL-C control in AMI populations. We also found the linear relationship of LDL-C levels with baseline SUA using the smooth curve fitting. The study raises the possibility that SUA may serve as a simple and noninvasive measurement to identify first AMI adults whose LDL-C was not being well controlled and a novel therapeutic evidence to formulate suitable individualized lipid-lowering strategies in early stage. Overall, the current studies are just hypothesis-generating, and further investigations are necessary to consolidate the results of this study.

The exact mechanisms by which the baseline SUA could predict the LDL-C were unclear, but it is biologically plausible. First, a higher level of SUA was generally associated with modern unhealthy lifestyles and diet rich in UA-raising components[13]. However, the cause of hyperlipidemia also includes genes and secondary factors in addition to poor diet and living habits. Thus, people with higher SUA levels after AMI may have better lipid control due to improvements in lifestyle and diet habits, compared with people having no bad habits to correct. Second, several studies reported that SUA can modulate LDL-C levels through enzymology. Minami et al. proposed that higher SUA levels significantly correlate with increased lipid peroxidation rates which are ameliorated by the xanthine oxidase inhibitor, allopurinol[14] and a critical role for SUA in inhibiting lipoprotein lipase activity in endothelial cells has also been suggested[15]. Further, it has been speculated that statins can lower serum creatinine levels by increasing renal blood flow and renal urate excretion[16–18]. Patients with higher uric acid may get greater lipid-lowering benefits from statin lipid-lowering therapy due to the uric acid-lowering effect of statins. Third, previous study showed that the reduction rate of small dense LDL (sdLDL) in patients with ACS with metabolic syndrome after 6 months of statin treatment was 5.5 times that of patients with ACS without metabolic syndrome[19]. Moreover, patients with metabolic syndrome used to behaved as hyperuricmia[20], and previous studies showed that small dense LDL-C was significantly associated with SUA levels[21, 22]. Therefore, it can be speculated that patients with higher sdLDL level used to behave as higher SUA and have higher decrease of LDL-C after statins therapy. Due to the increasing number of studies with respect to association between SUA and LDL-C, it is reasonable to use SUA to assess the condition of lipid control. The results only suggested that first AMI patients with higher SUA had a greater reduction in LDL-C and were more likely to achieve LDL-C goal.

### Study strengths and limitations

This study is currently the first study to assess the predictive value of baseline SUA with the change of LDL-C level in AMI patients underlying statins therapy. Nevertheless, several potential limitations of this study are noteworthy. First, as a single-center, retrospective observational study, residual confounding or selection bias cannot be excluded, which is inherent to any retrospective study. Second, the patients' adjusted dose of statins or diuretics, which may affect blood lipid variability. Third, the study population was from first-AMI patients in southern China. Therefore, these results cannot be generalized to other race groups, regions, or types of diseases.

## Conclusion

Among Chinese patients with first AMI underlying statins lipid-lowering therapy, percentage decrease of LDL-C (%) was independently and positively associated with baseline SUA. Besides, the rate of LDL-C contol at 6 months follow-up was significantly related to baseline SUA. Therefore, the baseline SUA acquired when AMI occurs, which are inexpensive and universally used in clinical practice, may improve the risk stratification of LDL-C uncontrol and select favourable candidates for aggressive lipoprotein-lowering therapy.

## Supplementary Information

**Additional file 1: Supplemental Figure 1.** The association between baseline SUA and LDLC control in various subgroups.

## Data Availability

The datasets used and/or analyzed during the current study are available from the corresponding author on reasonable request.

## Abbreviations

SUA: Serum uric acid
TC: Total cholesterol
TG: Triglyceride
HDL-C: High-density lipoprotein cholesterol
LDL-C: Low-density lipoprotein cholesterol
non-HDL-C: Non-high-density lipoprotein cholesterol
ApoA: Apolipoprotein A
ApoB: Apolipoprotein B
ATF: Atorvastatin
ROS: Rosuvastatin
BP: Blood Pressure
SBP: Systolic blood pressure
DBP: Diastolic blood pressure
AMI: Acute myocardial infarction
STEMI: ST-segment elevation myocardial infarction
NSTEMI: Non-ST-segment elevation myocardial infarction
ASCVD: Atherosclerotic cardiovascular disease
BMI: Body mass index
BUN: Blood urea nitrogen
CRE: Creatinine
eGFR: Estimated glomerular filtration rate
OR: Odd ratio
CI: Confidence interval
SD: Standard deviation
sdLDL: Small dense LDL
TBil: Total bilirubin
DBIL: Direct bilirubin
IBIL: Indirect bilirubin
ALT: Alanine aminotransferase
AST: Aspartate aminotransferase
LAD: Left anterior descending coronary
RCA: Right coronary artery
LCX: Left circumflex branch
LM: Left main coronary artery
LVEF: Left ventricular ejection fraction
