# Supplementary material for: Association between baseline serum uric acid and development of LDL-C level in patients with first acute myocardial infarction
Source: BMC Cardiovasc Disord. 2021 Nov 30;21:572. doi: 10.1186/s12872-021-02383-x (PMC8638344; doi:10.1186/s12872-021-02383-x)
Supplement: Supplementary file 1 — Additional file 1: Supplemental Figure 1. The association between baseline SUA and LDLC control in various subgroups. [file 12872_2021_2383_MOESM1_ESM.pdf]

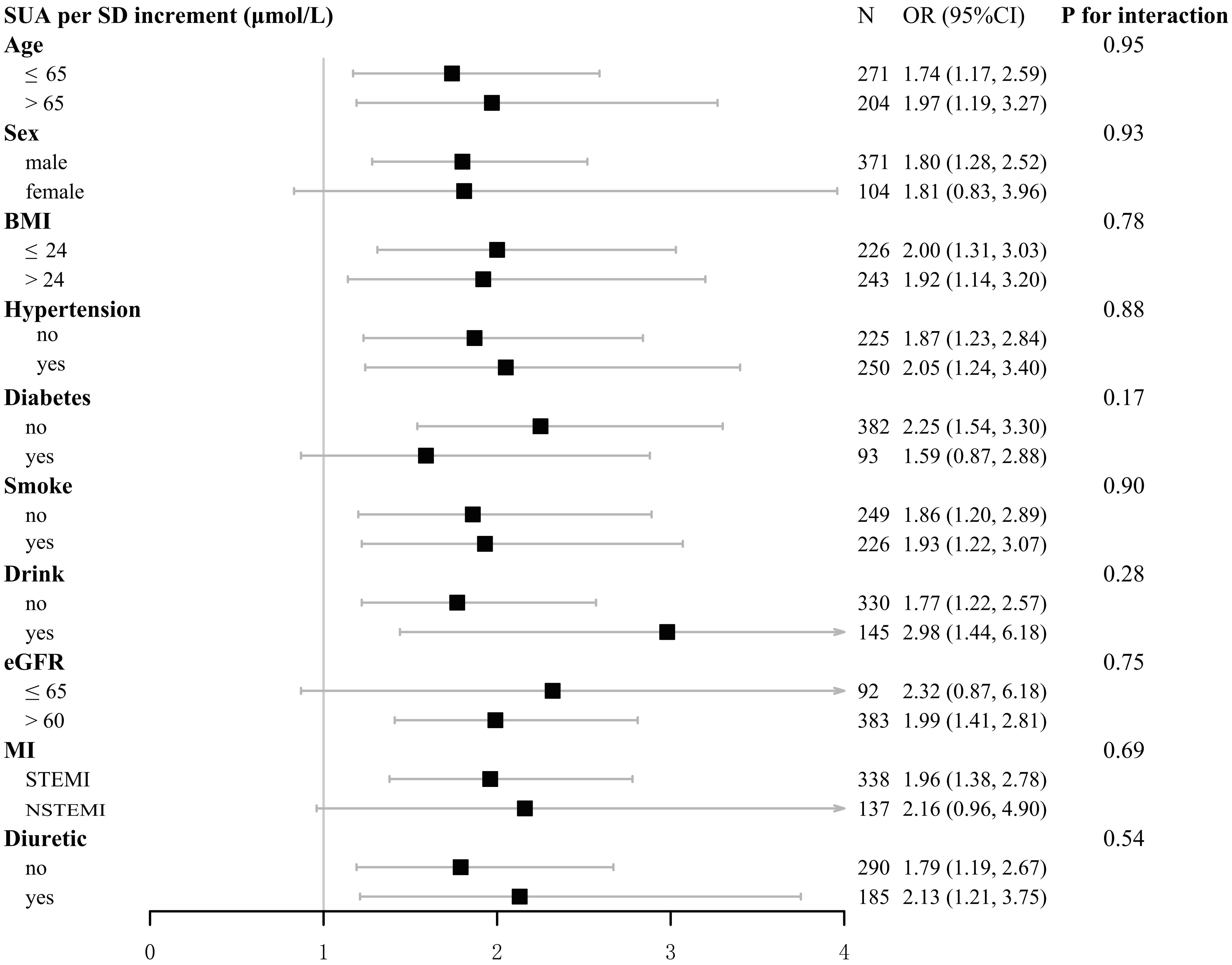

Supplemental Figure 1. The association between baseline SUA and LDLC control in various subgroups*.
*Adjusted, if not stratified, for sex, BMI, age, hypertension, diabetes, smoke, drink, types of AMI, gout, TG, LDL-C, BUN, CRE, eGFR, AST, ALT, TBIL, statin use, uric acid lowering drugs, diuretics and β receptor blocker.
